# GDF-15 in tumor-derived exosomes promotes muscle atrophy via Bcl-2/caspase-3 pathway

**DOI:** 10.1038/s41420-022-00972-z

**Published:** 2022-04-04

**Authors:** Wanli Zhang, Weikuan Sun, Xiaofan Gu, Chunxiao Miao, Lixing Feng, Qiang Shen, Xuan Liu, Xiongwen Zhang

**Affiliations:** 1 Shanghai Engineering Research Center of Molecular Therapeutics and New Drug Development, School of Chemistry and Molecular Engineering, East China Normal University, Shanghai, China; 2 Institute of Interdisciplinary Integrative Medicine Research, Shanghai University of Traditional Chinese Medicine, Shanghai, China

**Keywords:** Cancer metabolism, Cancer models

## Abstract

Tumor-derived exosomes are emerging mediators of cancer cachexia, a kind of multifactorial syndrome characterized by serious loss of skeletal muscle mass and function. Our previous study had showed that microRNAs in exosomes of C26 colon tumor cells were involved in induction of muscle atrophy. Here, we focus on studying proteins in tumor-derived exosomes which might also contribute to the development of cancer cachexia. Results of comparing the protein profiles of cachexic C26 exosomes and non-cachexic MC38 exosomes suggested that growth differentiation factor 15 (GDF-15) was rich in C26 exosomes. Western blotting analysis confirmed the higher levels of GDF-15 in C26 cells and C26 exosomes, compared with that of MC38 cells. Results of animal study also showed that GDF-15 was rich in tumor tissues, serum exosomes, and gastrocnemius (GA) muscle tissues of C26 tumor-bearing mice. GDF-15 protein could directly induce muscle atrophy of cultured C2C12 myotubes via regulating Bcl-2/caspase-3 pathways. What’s more, overexpression of GDF-15 in MC38 cells could increase the potency of MC38 conditioned medium or exosomes in inducing muscle atrophy. Knockdown of GDF-15 in C26 cells decreased the potency of C26 conditioned medium or exosomes in inducing muscle atrophy. These results suggested that GDF-15 in tumor-derived exosomes could contribute to induction of muscle atrophy and also supported the possibility of targeting GDF-15 in treatment of cancer cachexia.

## Introduction

Cancer cachexia is responsible for death of more than 20% of cancer patients directly and indirectly [1]. Colorectal cancer (CRC) has become the third most common malignancy cancer and the second leading cause of cancer mortality worldwide [2]. Notably, cancer cachexia affects around 50–61% of colorectal cancer patients but remains understudied and uncured [3]. Cancer cachexia has been defined as a multifactorial syndrome including ongoing loss of muscle mass (with or without loss of fat) that cannot be completely reversed by conventional nutritional support and leads to progressive functional impairments [4]. Cancer cachexia decreases quality of life and tolerance of antitumor treatment [5]. Thus, it is urgent to investigate the molecular mechanisms of cancer cachexia to promote the discovery of potential therapeutic targets and the development of new drugs [1].

Exosomes had been reported to be involved in the progression of cancer cachexia [6, 7]. Exosomes are now considered as an integral part of the intercellular microenvironment and may act as regulators of cell-to-cell communication [8]. Cargos in exosomes contain functional nucleic acids as well as proteins which can be absorbed and influence the biological functions of recipient cells [9–12]. Previous studies showed that IL-6, HSP70/90 and miRNAs in cancer exosomes could participate in triggering muscle degradation [6, 13–16], emphasizing the important roles of cancer exosomes in the development of cancer cachexia. In our previous study, we also found that miR-195a-5p and miR-125b-1-3p in exosomes of C26 colon tumor cells could induce muscle atrophy by apoptosis [6]. However, the roles of proteins in tumor-derived exosomes in the development of muscle atrophy in cancer cachexia have not been fully clarified. This inspired us to focus on studying the functional proteins in C26 exosomes which might be active in inducing muscle atrophy.

In the present study, we firstly compared the protein profiles of C26 exosomes and exosomes of non-cachexic MC38 tumor cells, which exhibited relatively weak potency in inducing cancer cachexia, to search possible active proteins in tumor-derived exosomes. Interestingly, growth differentiation factor 15 (GDF-15) was found to be rich in C26 exosomes, compared with MC38 exosomes. GDF-15 is a distant member of the transforming growth factor beta (TGF-β) superfamily which was initially named as macrophage inhibitory cytokine-1 [17]. GDF15 is expressed at a low concentration in multiple tissues in normal physiology but its expression could be induced by mitochondrial dysfunction, cellular stress, inflammation, aging or other pathological conditions thus it is considered as a stress response cytokine [18]. GDF15 was reported to play a role in regulating inflammatory and apoptotic pathways in injured tissues and during disease processes [19, 20]. Notably, GDF-15 had been reported to play roles in cancer cachexia. The expression of GDF15 increased in patients with gastric, pancreatic, prostate, colorectal and melanoma [21–25]. Elevation in circulating GDF15 correlates with cancer cachexia [26] and GDF15-induced weight loss in mice was reported to be mainly mediated by a GDNF family receptor alpha like (GFRAL)-Ret proto-oncogene (RET) signaling complex in brainstem neurons [27]. Blocking GDF15 signaling could reverse cachexia [28]. A therapeutic antagonistic monoclonal antibody, which targets GFRAL and inhibits RET signaling by preventing the GDF15-driven interaction of RET with GFRAL on the cell surface, successfully prevented cancer cachexia [29]. Previous studies also suggested that increased expression of GDF-15 may mediate muscle atrophy in pulmonary arterial hypertension and intensive care unit-acquired weakness patients [30, 31]. GDF-15 elevated levels are associated with body weight loss in numerous chronic human diseases and an increased risk of recurrence and reduced over survival [32, 33]. In the present study, to study the roles of GDF-15 in cancer cachexia, the levels of GDF-15 in C26 cells and C26 exosomes were detected and compared with that of MC38 cells. Animal study checking the levels of GDF-15 in tumor tissues, serum exosomes, muscle tissues of C26-tumor bearing mice was also conducted. Furthermore, the direct atrophy-inducing effects of GDF-15 on cultured C2C12 myotubes were checked and the possible mechanisms of GDF-15 were clarified. The influence of changes in GDF-15 expression in C26 or MC38 cells on the potency of tumor cells in inducing muscle atrophy was also observed. Results of the present study suggested that GDF-15 contained in tumor-derived exosomes might be involved in muscle wasting in cancer cachexia via inducing apoptosis of myocytes. These findings shed lights on understanding the mechanism of cancer cachexia and developing novel strategies for the treatment of cancer cachexia.

## Results

### Cachectic C26 exosomes contain high level of GDF-15

**Fig. 1:**
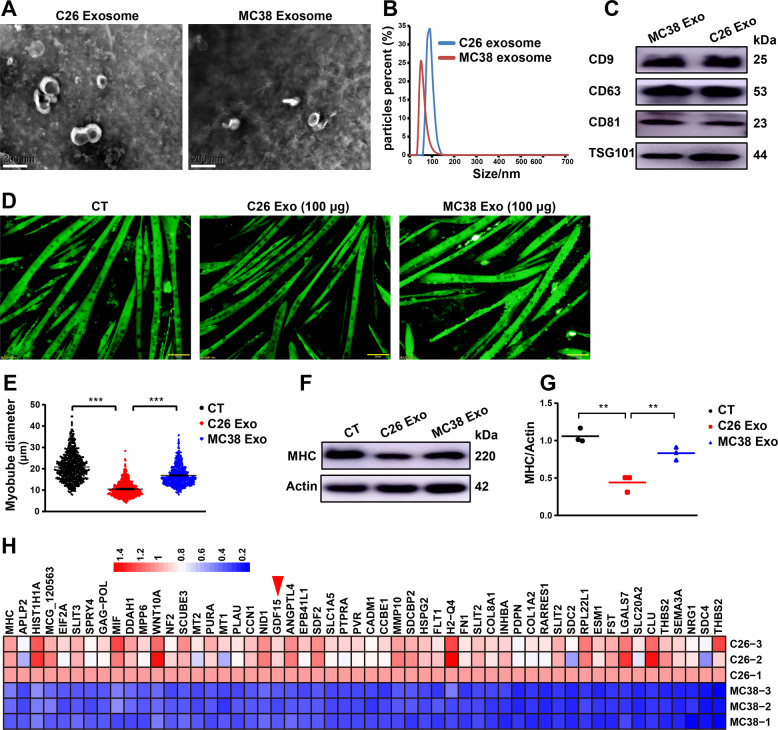
Cachectic C26 exosomes contain high level of GDF15. **A** Representative transmission electron microscopy image of C26 and MC38 exosomes (scale bars, 200 nm). **B** Particle size analysis of exosomes derived from C26 and MC38 cells. **C** Western blots showed the expression of exosome markers (CD9, CD63, CD81, TSG101). **D** Representative immunofluorescent images of C2C12 myotubes treated with exosomes (scale bars, 50 μm). The C2C12 myotubes wasting were induced by exosomes (100 μg) of C26 and MC38 respectively for 48 h. **E** Quantification of diameter of C2C12 myotubes treated with exosomes (*n* = 653,653,653, ***p < 0.001; *t* test). **F**, **G** Western blot analysis of MHC (*n* = 3, **p < 0.01; *t* test). **H** Proteomics was performed to figure out the difference in protein profiles of C26 exosomes versus MC38 exosomes. Data presented are the mean ± SEM.

Exosomes of C26 or MC38 cells were isolated from culture medium. As shown in Fig. 1A, B, electron microscopy and Zeta nanoparticle tracking analyzer showed that isolated vesicles were exosomes with cup-shaped morphology and a diameter of 30–150 nm. Exosomes were also verified by the exosome specific protein markers, CD9, CD63, CD81, and TSG101 (Fig. 1C). Compared with non-cachexia MC38 exosomes, cachexic C26 exosomes were able to induce C2C12 myotube atrophy thus the diameter of myotubes and the protein level of MHC were significantly decreased in C26 exosomes-treated C2C12 myotubes (Fig. 1 D–G). Quantitative proteomic analysis was used to compare the protein profiles of C26 exosomes and MC38 exosomes. Overall, 1599 proteins were detected. Among them, 95.1% proteins maintained constant level in both C26 and MC38 exosomes; 3.4% (54 proteins) were significantly higher in C26 exosomes than in MC38 exosomes; 1.5% (24 proteins) were significantly lower in C26 exosomes than in MC38 exosomes. The 53 proteins, including GDF-15, with higher levels in C26 exosomes were shown in Fig. 1H. Together, these data demonstrated the capability of C26 exosomes in inducing muscle atrophy and the possible important protein GDF-15 in C26 exosomes.

### Confirmation of high level of GDF-15 in C26 cells, C26 exosomes and secretion of GDF-15 via exosomes

**Fig. 2:**
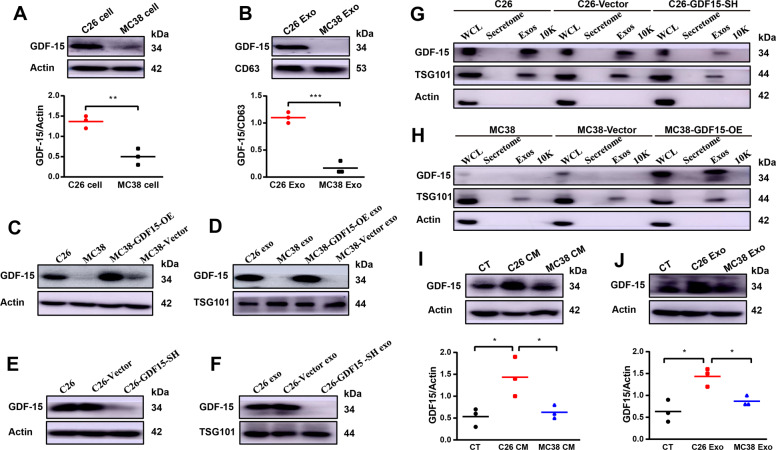
Confirmation of high level of GDF15 in C26 cells, C26 exosomes and secretion of GDF15 via exosomes. The stable cell lines of MC38-GDF15-OE, MC38-Vector, C26-GDF15-SH, C26-Vector were constructed by infected with corresponding lentivirus and then monoclonal cells were picked out for the further study. **A** Comparison of GDF15 expression levels in MC38 cells and C26 cells (*n* = 3, *p < 0.01; *t* test). **B** Comparison of GDF15 levels in MC38 exosomes and C26 exosomes (*n* = 3, ***p < 0.001; *t* test). **C**, **D** Western blot analysis to confirm the up-regulation of GDF15 expression in MC38-GDF15-OE cells **C** and exosomes **D**. **E**, **F** Western blot analysis to confirm the knockdown of GDF15 expression in C26-GDF15-SH cells **E** and exosomes **F**. **G** Western blot analysis of GDF15 level in WCL (whole cell lysates), secretome (soluble secreted proteins), Exos (exosomes) and the cell medium pellet obtained after 10,000 g (10 K) derived from C26 cells, C26-Vector cells and C26-GDF15-SH cells respectively. **H** Western blot analysis of GDF15 level in WCL, secretome, Exos and 10 K derived from MC38 cells, MC38-Vector cells and MC38-GDF15-SH cells respectively. **I** Western blot analysis of GDF15 level in C2C12 myotubes treated with C26 tumor cell medium (C26 CM) or MC38 tumor cell medium (MC38 CM) (*n* = 3, *p < 0.05; *t* test). **J** Western blot analysis of GDF15 level in C2C12 myotubes treated with C26 tumor cell exosomes (C26 Exo) and MC38 tumor cell exosomes (MC38 Exo) (*n* = 3, **p < 0.05; *t* test). Data presented are the mean ± SEM of three independent experiments.

Based on the results mentioned above, GDF-15 was selected for further study. Western blot results confirmed the significantly high levels of GDF-15 in C26 cells and exosomes compared to MC38 cells and exosomes, respectively (Fig. 2A, B). As shown in Fig. 2C, D, MC38 cell line with overexpression of GDF-15 (MC38-GDF15-OE) was established. As shown in Fig. 2E, F, C26 cells with shRNA knockdown of GDF-15 (C26-GDF15-SH) was also established. To check whether GDF-15 in C26 cells are released by exosomes or by direct secretion into culture medium, the amounts of GDF-15 in whole cell lysate (WCL), secretome (soluble secreted proteins), Exos (exosomes) and 10 K pellet (the cell medium pellet obtained after 10,000 g centrifugation) were analyzed and compared as previously described [34]. As shown in Fig. 2G, H, results of western blot suggested that GDF-15 was enriched in exosomes compared to the 10 K pellet and secretomes, indicating that GDF-15 was secreted from tumor cells mainly through exosomes. Results of wild type C26 cells, C26-GDF15-SH cells, wild MC38 cells and MC38-GDF15-OE cells all supported the release of GDF-15 from C26 cells through exosomes. Furthermore, as shown in Fig. 2I, J, the levels of GDF-15 in C2C12 myotubes treated with C26 conditioned medium or C26 exosomes were significantly higher than that of C2C12 myotubes treated with MC38 conditioned medium or MC38 exosomes. Collectively, these results verified that GDF-15 in C26 cells could be released and transported to recipient cells (C2C12 myotubes) by C26 exosomes.

### High level of GDF-15 in C26 tumor tissues, serum exosomes and GA muscle tissues of C26 tumor-bearing mice

**Fig. 3:**
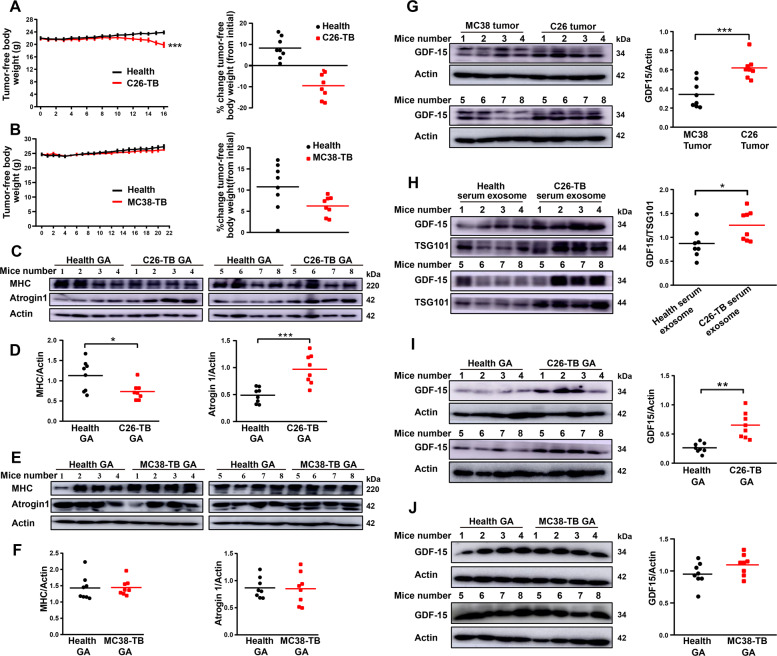
High level of GDF15 in C26 tumor tissues, serum exosomes and GA muscle tissues of C26 tumor-bearing mice. C26 tumors cells (1 × 10^6^) or MC38 tumor cells (1 × 10^6^) were inoculated subcutaneously in the right flank of BALB/c or C57BL/6 J mice respectively with 100 μL. Body weight and tumor volume were recorded every day until the end of experiment. **A** Time-related change in tumor-free body weight of the C26-bearing mice (*n* = 8). **B** Time-related change in tumor-free body weight of the MC38-bearing mice (*n* = 8). **C**, **D** Western blot analysis of MHC and Atrogin1 protein in GA tissues in C26-bearing mice compared to health group (*n* = 8, *p < 0.05, ***p < 0.001; *t* test). **E**, **F** Western blot analysis of MHC and Atrogin1 protein of GA tissues in MC38-bearing mice versus health group (*n* = 8). **G** Western blot analysis of GDF15 levels in MC38 tumor and C26 tumor tissues (*n* = 8, ***p < 0.001; *t* test). **H** Western blot analysis of GDF15 content in serum exosome of C26-bearing mice versus health group (*n* = 8, *p < 0.05; *t* test). **I** Western blot analysis of GDF15 protein levels in GA tissues of C26-bearing mice in comparison with health group (*n* = 8, **p < 0.01; *t* test). **J** Western blot analysis of GDF15 protein levels in GA tissues of MC38-bearing mice compared to health group (*n* = 8). The data presented are the mean ± SEM.

To investigate the role of GDF-15 in skeletal muscle atrophy of cancer cachexia in vivo, tumor-bearing mice models were established by subcutaneous implantation of two mouse cell lines (C26 and MC38) into BALB/c or C57BL6/ mice, respectively. MC38 is a kind of non-cachectic cancer cell line and can be used as a negative control in comparison with C26 cell line [6]. As shown in Fig. 3A, in comparison with the body weight of non-tumor-bearing mice (Health group), mice implanted with C26 cells showed significant decrease in tumor-free body weight at the end of the experiment. While, mice inoculated with MC38 cells did not induce significant decrease in tumor-free body weight at the end of the experiment (Fig. 3B). Furthermore, the protein levels of skeletal muscle atrophy marker MHC was significantly decreased and Atrogin 1 was significantly up-regulated in GA muscle tissues of C26-bearing mice (Fig. 3C, D). In contrast, there was no difference in the protein levels of MHC and Atrogin 1 in GA muscle tissues between MC38-bearing mice and non-tumor-bearing mice (Fig. 3E, F). These results confirmed the potency of C26 tumor cells but not MC38 tumor cells in inducing muscle atrophy. Notably, the level of GDF-15 in C26 tumor tissues was significantly higher than that of MC38 tumor tissues (Fig. 3G). Likewise, the level of GDF-15 was higher in C26-bearing mice serum exosomes than health mice serum exosomes (Fig. 3H). What’s more, the concentration of GDF-15 in GA tissues of C26-bearing mice was significantly higher than that of health mice (Fig. 3I). On the contrary, there was no difference in GDF-15 protein level in GA tissues between MC38-bearing mice and health mice (Fig. 3J). Together, these results confirmed that GDF-15, possibly as an active component in exosomes, was involved in the development of muscle atrophy in cancer cachexia.

### GDF-15 could induce muscle atrophy both extracellularly and intracellularly

**Fig. 4:**
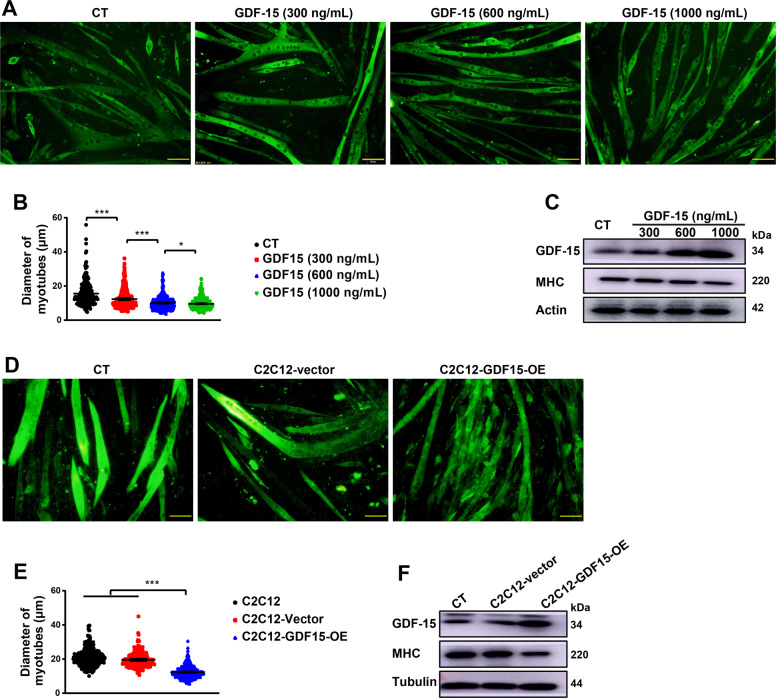
GDF15 could induce muscle atrophy both extracellularly or intracellularly. C2C12 myotubes differentiated from myoblasts were treated with recombinant GDF15 (final concentration:300 ng/mL, 600 ng/mL, 1000 ng/mL) for 48 h, or over-expressed with GDF15 by infected with corresponding lentivirus for 72 h. **A** Representative immunofluorescent images of C2C12 myotubes treated with recombinant GDF15 protein in different concentrations (scale bars, 50 μm). **B** Quantification analysis of C2C12 myotubes diameter (*n* = 240,391,474,652, *p < 0.05, ***p < 0.001; *t* test). **C** Western blot analysis of GDF15 and MHC expression in myotubes. **D** Representative immunofluorescent images of C2C12-GDF15-overexpression myotubes (scale bars, 50 μm). **E** Quantification analysis of C2C12 myotubes diameter (*n* = 334,315,437, ***p < 0.001; *t* test). **F** Detection of GDF15 and MHC protein level in C2C12-GDF15-overexpression myotubes (scale bars, 50 μm). Data presented are the mean ± SEM.

In order to investigate the functions of GDF-15 in skeletal muscle atrophy, recombinant GDF-15 protein was added to the medium of C2C12 myotubes at different final concentrations (300 ng/mL, 600 ng/mL, 1000 ng/mL). As shown in Fig. 4A, B, extracellular GDF-15 dose-dependently induced muscle atrophy of C2C12 myotubes. The level of GDF-15 in C2C12 myotubes was increased by extracellular GDF-15 while, at the same time, the protein level of MHC was decreased, compared with control group (Fig. 4C). The influence of overexpression of GDF-15 in C2C12 myotubes was also observed. C2C12 myotubes were infected by lentivirus-encoding GDF-15. After 72 h of infection, the myotubes with GDF-15 overexpression were significantly thinner than those infected by vector virus (Fig. 4D, E). Consistently, the protein level of MHC was down-regulated while GDF-15 was up-regulated respectively in myotubes with overexpression of GDF-15, compared with control myotubes (Fig. 4F). Collectively, the results suggested that GDF-15 could directly induce muscle atrophy both extracellularly and intracellularly.

### GDF-15 induced muscle atrophy via up-regulating Bcl-2/caspase-3 pathway

**Fig. 5:**
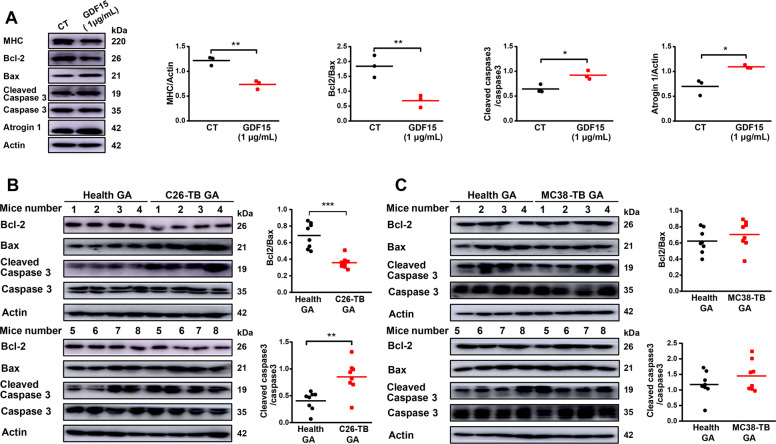
GDF15 induced muscle atrophy via up-regulating Bcl-2/caspase-3 pathway. **A** Detection of the protein level of MHC, Bcl-2, Bax, Cleaved caspase-3, Atrogin1 in C2C12 myotubes treated with recombinant GDF15 protein via western blot. C2C12 myotubes were treated with recombinant GDF15 (1 μg/mL) for 72 h (*n* = 3, *p < 0.05, **p < 0.01; *t* test). **B** Analysis of the protein level of Bcl-2, Bax, Cleaved caspase-3 in C26-bearing mice GA tissues versus health group via western blot (n = 8, **p < 0.01, ***p < 0.001; *t* test). **C** Analysis of the protein level of Bcl-2, Bax, Cleaved caspase-3 in MC38-bearing mice GA tissues compared to health group via western blot (*n* = 8). The data presented are the mean ± SEM.

As shown in Fig. 5A, treatment of GDF-15 induced significant decrease in the ratio of Bcl-2/Bax and significant increase in the ratio of cleaved caspase-3/caspase-3 in cultured C2C12 myotubes. At the same time, the level of MHC decreased significantly and the level of Atrogin1 increased dramatically in GDF-15 treated myotubes compared with control myotubes (Fig. 5A). Additionally, significant decrease in the ratio of Bcl-2/Bax and significant increase in the ratio of cleaved caspase-3/caspase-3 were also detected in GA muscle tissues of C26 tumor-bearing mice (Fig. 5B) compared with health mice. On the contrary, there was no difference in the activation of apoptotic pathways in GA tissues from MC38-bearing mice compared with health mice (Fig. 5C). Overall, these data suggested that muscle atrophy in cancer cachexia was related to activation of apoptosis while GDF-15 might induce apoptosis in muscle tissues via activating Bcl-2/caspase-3 pathway.

### Contribution of GDF-15 in the potency of tumor cells and tumor exosomes in inducing muscle atrophy

**Fig. 6:**
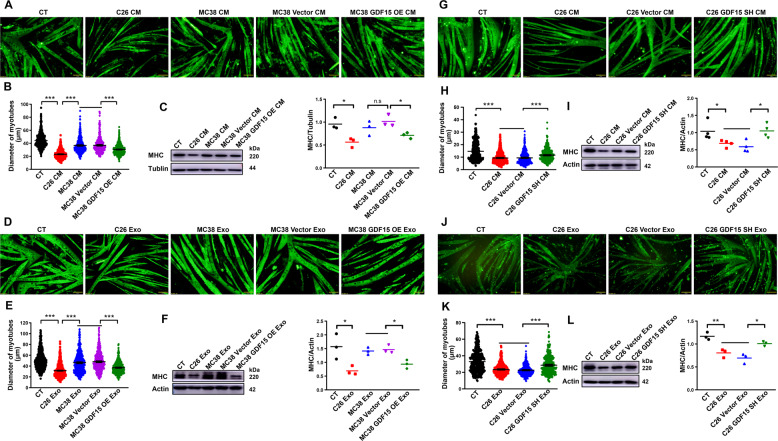
Contribution of GDF15 in the potency of tumor cells and tumor exosomes in inducing muscle atrophy. C2C12 myotubes were treated with MC38-GDF15-OE cell medium, exosomes or C26-GDF15-SH cell medium, exosomes respectively. **A** Representative immunofluorescent images of C2C12 myotubes treated with MC38-GDF15-OE cell culture medium (scale bars, 50 μm). **B** Quantification analysis of C2C12 myotubes diameter (*n* = 546,524,508,401,540, ***p < 0.001; *t* test). **C** MHC protein expression analysis via western blot (*n* = 3, ns *p* > 0.05, *p < 0.05; *t* test). **D** Representative immunofluorescent images of C2C12 myotubes treated with MC38-GDF15-OE cell exosomes (scale bars, 50 μm). **E** Quantification analysis of C2C12 myotubes diameter (*n* = 662,960,544,507,507, ***p < 0.001; *t* test). **F** MHC protein expression analysis via western blot (*n* = 3, *p < 0.05; *t* test). **G** Representative immunofluorescent images of C2C12 myotubes treated with C26-GDF15-SH cell culture medium (scale bars, 50 μm). **H** Quantification analysis of C2C12 myotubes diameter (*n* = 564,539,472,445, ***p < 0.001; *t* test). **I** MHC protein expression analysis via western blot (*n* = 4, *p < 0.05; *t* test). **J** Representative immunofluorescent images of C2C12 myotubes treated with C26-GDF15-SH cell exosomes (scale bars, 50 μm). **K** Quantification analysis of C2C12 myotubes diameter (*n* = 361,359,361,367, ***p < 0.001; *t* test). **L** MHC protein expression analysis via western blot (*n* = 3, *p < 0.05, **p < 0.01; *t* test). Data presented are the mean ± SEM.

To check the contribution of GDF-15 to induction of muscle atrophy by tumor cells, the influence of changes in GDF-15 expression on the potency of tumor cells, especially tumor exosomes, in inducing muscle atrophy was observed. As shown in Fig. 6A, B, the conditioned medium of MC38-GDF15-OE cells, but not MC38-GDF15-Vector cells, could significantly decrease myotubes diameter of C2C12 myotubes. The protein level of MHC in C2C12 myotubes treated with MC38-GDF15-OE medium, but not MC38-GDF15-Vector medium, was also significantly decreased (Fig. 6C). More importantly, we also observed that MC38-GDF15-OE exosomes could significantly reduce myotubes diameter, in comparison with MC38-GDF15-Vector exosomes (Fig. 6D, E). The protein level of MHC also significantly lower in C2C12 myotubes treated with MC38-GDF15-OE exosomes, but not MC38-GDF15-Vector exosomes (Fig. 6F). Furthermore, knockdown of GDF-15 expression in C26 cells significantly alleviated the potency of cell conditioned medium (C26-GDF15-SH medium) in inducing muscle atrophy of C2C12 myotubes, compared with C26-Vector medium (Fig. 6G, H). The protein level of MHC in C26-GDF15-SH medium, but not C26-Vector medium, treated myotubes was also significantly increased (Fig. 6I). More importantly, the data also showed that C26-GDF15-SH exosomes exhibited significantly alleviated potency in decreasing the diameter of C2C12 myotubes, compared with C26-Vector exosomes (Fig. 6J, K). The protein level of MHC in C2C12 myotubes treated with C26-GDF15-SH exosomes, but not C26-Vector exosomes, was significantly increased (Fig. 6L). These results suggested that changes in GDF-15 expression level would result in corresponding changes in the potency of tumor cells, especially tumor exosomes, in inducing muscle atrophy.

### Confirmation of the involvement of apoptotic pathway in GDF-15 induced muscle atrophy

**Fig. 7:**
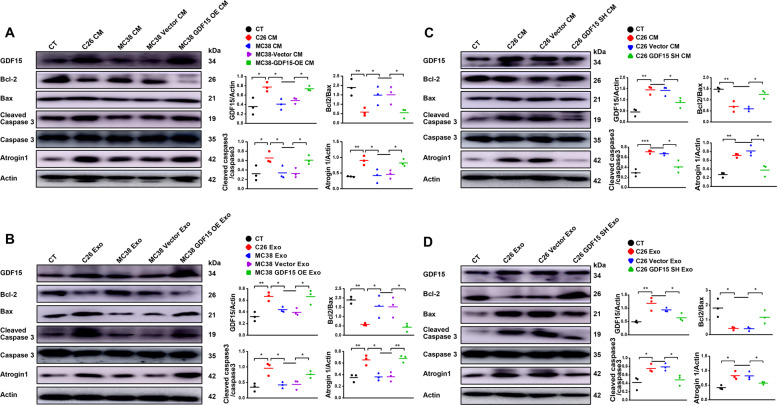
Confirmation of the involvement of apoptotic pathway in GDF15-induced muscle atrophy. C2C12 myotubes were treated with MC38-GDF15-OE cell medium, exosomes or C26-GDF15-SH cell medium, exosomes respectively. **A** Analysis of the protein level of MHC, GDF15, Bcl-2, Bax, Cleaved caspase-3, Atrogin1 in C2C12 myotubes treated with MC38-GDF15-OE cell medium (*n* = 3, *p < 0.05, **p < 0.01; *t* test). **B** Analysis of the protein level of MHC, GDF15, Bcl-2, Bax, Cleaved caspase-3, Atrogin1 in C2C12 myotubes treated with MC38-GDF15-OE cells derived exosomes (*n* = 3, *p < 0.05, **p < 0.01; *t* test). **C** Analysis of the protein level of MHC, GDF15, Bcl-2, Bax, Cleaved caspase-3, Atrogin1 in C2C12 myotubes treated with C26-GDF15-SH cell medium (*n* = 3, *p < 0.05, **p < 0.01, ***p < 0.001; *t* test). **D** Analysis of the protein level of MHC, GDF15, Bcl-2, Bax, Cleaved caspase-3, Atrogin1 in C2C12 myotubes treated with C26-GDF15-SH cells derived exosomes (*n* = 3, *p < 0.05, **p < 0.01; *t* test). Data presented are the mean ± SEM.

**Fig. 8:**
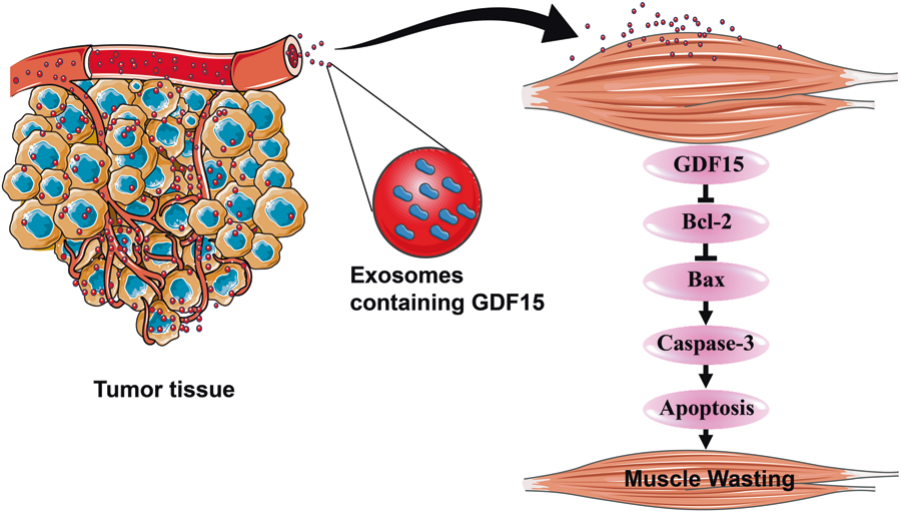
Illustration of the mechanisms of exosome GDF15 in inducing muscle atrophy. Exosomes derived from cachexic C26 cells containing abundant GDF15 which could induce muscle wasting via activating Bcl-2/caspase-3-based apoptosis pathway in myocytes.

The activation of apoptotic pathways were observed in C2C12 myotubes treated with conditioned medium or exosomes of C26 cells or MC38 cells, with or without overexpression of GDF-15 or knockdown of GDF-15 expression respectively. As shown in Fig. 7A, C2C12 myotubes treated with C26 medium or MC38-GDF15-OE medium, but not MC38 medium or MC38 vector medium, exhibited decrease in ratio of Bcl-2/Bax, increase in Atrogin 1 and cleaved caspase-3/caspase-3. Similarly, as shown in Fig. 7B, C2C12 myotubes treated with C26 exosomes or MC38-GDF15-OE exosomes, but not MC38 exosomes or MC38 vector exosomes, exhibited decrease in ratio of Bcl-2/Bax, increase in Atrogin 1 and cleaved caspase-3/caspase-3. These results confirmed the increase in the potency of MC38 conditioned medium and MC38 exosomes in inducing muscle atrophy by overexpression of GDF-15 in MC38 cells and also confirmed the involvement of apoptotic pathway in GDF-15 induced muscle atrophy. On the other hand, as shown in Fig. 7C, D, knockdown of GDF-15 in C26 cells ameliorated the potency of C26 medium or C26 exosomes in inducing decrease in ratio of Bcl-2/Bax, increase in Atrogin 1 and cleaved caspase-3/caspase-3. These results confirmed the decrease in the potency of C26 conditioned medium and C26 exosomes in inducing muscle atrophy by knockdown of GDF-15 in C26 cells and also confirmed the involvement of apoptotic pathway in GDF-15 induced muscle atrophy. Fig. 8

## Discussion

Cancer cachexia is a poor prognostic indicator that can profoundly affect well-being and the ability to tolerate cancer treatments [5]. Cancer cachexia is highly associated with pancreatic cancer, gastro-esophageal cancer, head and neck cancers, lung cancer, colorectal cancer, hematological cancers, breast cancer, and prostate cancer [35]. Half of all cancer deaths worldwide (~8.2 million people per year) are attributed to the cancers most frequently associated with cachexia, namely, pancreatic (0.33 million deaths), esophageal (0.40 million), colorectal (0.69 million), gastric (0.72 million), hepatic (0.75 million) and pulmonary (1.59 million) [35]. Therefore, clarification of the mechanisms involved in cancer cachexia is indeed necessary for seeking new methods alleviating cachexia. Cancer exosomes were reported to regulate angiogenesis, metastasis, and chemo-resistance of tumor [36]. Exosomes also play important roles in both normal and abnormal physiology including normal maintenance and degeneration of musculoskeletal tissues [37]. More and more studies verified that exosomes derived from tumor cells were involved in the process of muscle wasting and fat lipolysis induced by cancer cachexia [38–42]. In line with these previous studies, our group had also verified that cancer exosomes contribute to the development of cancer cachexia [6]. The serum exosomes content in cachexia cancer patients was significantly higher than that of non-cachexia cancer patients and similar results were observed in serum of cachectic C26 bearing mice compared to non-cachectic MC38 bearing mice. Inhibition of exosome biogenesis of tumor cells would decrease the potency of tumor exosomes in inducing cancer cachexia both in vitro and in vivo [6].

Both proteins and nucleic acids in exosomes might be involved in the induction of skeletal muscle wasting [6, 15, 38, 43]. Here, this study focused on searching important proteins in C26 exosomes with potency of inducing muscle atrophy in cancer cachexia. Our results showed that GDF-15 might be an important protein in C26 exosomes which could contribute to induction of muscle atrophy. High level of GDF-15 was observed in C26 cells as well as C26 exosomes, in comparison with MC38 cells and exosomes respectively. The level of GDF-15 was significantly higher in C2C12 myotubes treated with C26 cell medium or exosomes than that in C2C12 myotubes treated with MC38 cell medium or exosomes respectively. What is more, the level of GDF-15 in serum exosomes and GA tissues of C26-bearing mice was dramatically higher versus health group. Our results suggested the possible important role of GDF-15 in muscle atrophy of cancer cachexia and were consistent to previous reports about GDF15 in cachexia. As a stress response protein, GDF-15 can be induced universally under the conditions of injury, inflammation and malignancy leading to modest increases in many disease processes and especially advanced cancers [19, 44]. Previous studies have verified that GDF-15 can cause cachexia and targeting GDF-15 in animal models rescued weight loss [45, 46]. Vickie E. Baracos et al. reported that pro-cachexia cytokine GDF-15 is sufficient to promote catabolism in skeletal muscle through tumor xenograft models [35]. Additionally, human epidemiology studies also indicated the correlation of serum levels of GDF-15 to markers of anorexia/cachexia syndromes through several diseases such as prostate cancer [44, 47], advanced pancreatic cancer [48], advanced esophageal squamous cell carcinoma [49], mixed populations of patients with cancer cachexia [46, 50, 51], chronic obstructive lung disease [52, 53] and intensive care associated cachexia [30]. Compared with the previous reports, our study not only confirmed the role of GDF-15 in cancer cachexia but also showed that GDF-15 could be secreted by tumor cells into tumor-derived exosomes and contributed to the high circulation level of GDF15 in cancer cachexia animals. More importantly, the delivery of GDF15 in exosomes made it to be easily absorbed by recipient cells and conduct regulating effects on various organs. Our results showed that GDF15 in exosomes could be absorbed by C2C12 myotubes and resulted in muscle atrophy.

The well-known receptor of GDF15 is GFRAL, a previously orphan member of the GFRα receptor family [27, 54]. The GFRAL is mainly localized in the central nervous system (CNS) and there is very limited evidence for its expression outside the CNS, exceptions being the testis and adipose tissue. GDF15 could acts as a critical mediator of anorexia-cachexia through the GDF15-mediated activation of hindbrain GFRAL-RET receptors [18]. It had also been showed that treatment with 3P10, a therapeutic antagonistic monoclonal antibody that targets GFRAL and inhibits RET signaling by preventing the GDF15-driven interaction of RET with GFRAL on the cell surface, reverses excessive lipid oxidation in tumor-bearing mice and prevents cancer cachexia, even under calorie-restricted conditions [29]. However, there is apparent contradiction between a cytokine capable of being expressed in most locations with apparent pleiotropic actions, and the highly localized receptor expression [54]. Thus, it is important and necessary to clarify the effects and mechanisms of GDF15 which are independent of GFRAL. Results of the present study showed that GDF-15 could directly induce muscle atrophy both extracellularly and intracellularly. Either treatment of C2C12 myotubes with recombinant GDF-15 protein or over-expression of GDF-15 in C2C12 myotubes could induce atrophy of C2C12 myotubes. Consistently, Bloch SA et al. have found that GDF-15 promote muscle wasting in intensive care unit-acquired weakness (ICUAW) through down-regulating the expression of muscle microRNAs [30]. GDF-15 might induce muscle atrophy via suppressing TAK1 in pulmonary arterial hypertension (PAH) [31]. Oba et al. demonstrated that elevated serum GDF-15 indicated low muscle strength and lower physical performance in older patients [55]. Kim et al. also confirmed loss of muscle mass and deteriorated muscle function accompanied with high levels of serum and muscle tissues GDF-15 [56]. In the present study, we further showed that overexpression of GDF-15 in non-cachexic MC38 cells could significantly increase the potency of both MC38 conditioned medium and MC38 exosomes in inducing muscle atrophy. On the contrary, knockdown of GDF-15 in cachexic C26 cells could significantly decrease the potency of both C26 conditioned medium and C26 exosomes in inducing muscle atrophy. These results suggested that GDF-15 contained in exosomes was sufficient and critical to promote muscle atrophy in cancer cachexia.

The apoptotic signaling is critical for controlling the protein degradation in muscle wasting, as well as the ubiquitin-proteasome and autophagy-lysosome pathway [39, 57]. Apoptosis activation has been found during cachexia of humans and mice. Busquets Sílvia *et al*. demonstrated that apoptosis is present in skeletal muscle of cachectic gastro-intestinal cancer patients [57, 58]. Apoptosis activation was observed in the development of cachexia in Apc (Min/+) mice [59]. To check whether GDF-15 also induce muscle atrophy by inducing apoptosis, we observed the activation state of apoptotic pathways in C2C12 myotubes treated with recombinant GDF-15, tumor cell medium, tumor-derived exosomes as well as in GA muscle tissues of tumor-bearing mice. Our results clearly showed that recombinant GDF-15 activated apoptotic pathways in C2C12 myotubes in comparison with control group. Similar results were also detected in GA tissues of C26-bearing mice versus health group. Apoptotic pathways were also activated in C2C12 myotubes treated with C26 medium, C26 exosomes, MC38-GDF15-OE medium or MC38-GDF15-OE exosomes respectively. On the contrary, activation of apoptotic pathways were not observed in C2C12 myotubes treated with MC36 medium, MC38 exosomes, C26-GDF15-SH medium or C26-GDF15-SH exosomes. Overall, these data demonstrated that GDF-15 could induce skeletal muscle wasting via activating apoptotic pathways. Interestingly, GDF15 had been reported to exhibit both pro-apoptotic effects [60–62] and anti-apoptotic effects [63–65]. By receiving a wide range of triggers and exerting a great number of downstream effects, GDF15 might play either positive or negative roles depending on the state of cells and its environment [18]. Further study is necessary to clarify the pathophysiological roles of GDF15 and try to develop drugs targeting GDF15 for the treatment of diseases such as cancer cachexia.

## Materials and methods

### Reagents

RIPA Lysis and Extraction Buffer and Halt Protease and Phosphatase Inhibitor Cocktail (100×) were purchased from Thermo Scientific and stored at 4 °C. BCA protein assay kit used to quantify protein concentration were purchased from Beyotime and stored at RT. DMEM (Hyclone, Logan, UT, USA), RPMI1640 (Hyclone, Logan, UT, USA), Penicillin/streptomycin and Trypsin/EDTA were purchased from Hyclone. Horse serum was purchased from Gibco (Gibco, Grand Island, NY, USA). Fetal bovine serum (FBS) was derived from Biological Industries, GW4869 (MCE, NJ, USA), PEG8000 (Sigma-Aldrich, MO, USA), X-treme GENE9 (Roche, Basel, Switzerland), Recombinant Mouse GDF15 (R & D, Minnesota, USA).

### Cell culture

C26 cells and MC38 cells were obtained from Shanghai Institute of Materia Medica, Chinese Academy of Sciences and maintained in RMPI-1640 medium containing 10% fetal bovine serum at 37 °C with 5% CO_2_. C2C12 murine myoblast cell line, obtained from ATCC (Manassas, USA), were cultured in high-glucose DMEM with 10% fetal bovine serum at 37 °C with 5% CO_2_. During differentiation, the medium of cells planted on culture plates coated with 0.1% gelatin was switched into differentiation medium (high-glucose DMEM containing 2% horse serum) when cell confluence reached 70%. After four days, multinuclear myotubes were formed. All cells were negative for mycoplasma contamination before use.

### Conditioned tumor medium collection and treatment on C2C12 myotubes

C26 or MC38 cells (2 × 10^6^) were seeded at 10 cm dish. The medium was switched to DMEM medium after 24 h later, and the cell culture medium was collected after 48 h. The standard operating procedure of cell medium collection and use was performed as previously described [6]. Briefly, the conditioned medium was collected and then the cells and debris were removed by centrifugation (1,000 × *g* for 5 min followed by 10,000 × *g* for 10 min). The supernatant was then filtered and used at a 1:1 dilution with fresh normal DMEM medium to treat C2C12 myotubes. Conditioned medium from non-tumor cells (C2C12 cells) was used as control medium. After 48 h treatment, the C2C12 myotubes were harvested for Western Blotting assay or used for morphological analysis.

### Exosomes isolation, quantitation, and characterization

As described before [6], exosomes were isolated from C26 and MC38 cells medium, mouse serum, respectively, using ExoQuick-TC Exosome Isolation Reagent (SBI, EXOTC10A-1) according to the manufacturer’s protocol. Briefly, culture supernatants were collected and centrifuged at 1,500 g for 5 min to pellet dead cells and cell debris. Then the Exosome Isolation Reagent was added to the supernatant and mixed up thoroughly. The mixture was then kept upright at 4 °C for 12 h and centrifuged at 1,500 × *g* for 30 min at 4 °C to pellet the exosomes. Then the supernatant was deserted and the white pellet containing exosomes at the bottom of the tube was suspended with PBS and quantified using BCA Protein Assay Kit (Beyotime, China). The characterization of exosomes were performed through transmission electron microscopy, particle size analysis and Western blotting analysis. The standard operating procedure was performed as previously described [6].

### Label-free proteomics analysis

Proteins contained in C26 and MC38 exosomes were extracted and the concentrations of proteins were detected by BCA Protein Assay Kit. Then the proteins were digested with trypsin to collect the peptides. After that, the peptides were extracted and prepared for analysis by the label free nanoLC-MS/MS approach. The mass spectrometer proteomics data were collected based on Thermo Scientific Q Exactive^TM^ BioPharma platform. Proteome Discoverer (v2.4) and Mascot (Matrix Science, London, UK, version 2.2) engine were used for the raw data processing and searching. And the whole progress of proteomics analysis was conducted at Shanghai Majorbio Biopharm Technology Co., Ltd (Shanghai, China). The screening conditions of significant changes were that the mean relative fold change of different genes (*P* < 0.01 and fold change>3).

### Plasmid construction and cell transfection

For the overexpression of GDF-15, the full-length coding region of gene GDF-15 has been cloned into PCDH-CMV-MCS-EF1-copGFP-T2A-Puro by XbaI-NotI. For the knockdown of GDF-15 expression, the short hairpin RNA (shRNA) of inhibiting GDF-15 expression has been constructed into PLKO.1-Puro-TRC by AgeI-EcoRI. And the following shGDF-15 sequence was used: 5’-GTGTCACTGCAGACTTATGAT-3’. All these plasmids were accomplished by Shanghai TSINGKE Biological Technology. C26 or MC38 cells were seeded at 6-well plates and transfected with corresponding lentivirus according to manufacturer’s protocol. Then the monoclonal cell lines of MC38-GDF15-OE, MC38-Vector, C26-GDF15-SH, C26-Vector were picked out for this research.

### Western blot

The assays were performed as previously described [6]. The primary antibodies used were as follows: GDF-15 (Abcam, ab128958), TSG101 (Proteintech, 28283-1-AP), Atrogin1 (Abcam, ab168372), CD9 (ABclonal, A19027), CD63 (ABclonal, A5271), CD81 (ABclonal, A5270), Bax (Proteintech, 50599-2-Ig), Bcl-2 (Proteintech, A11025), MHC (DSHB, MF20), Caspase3 (ABcolonal, A0214), Cleaved caspase3 (Cell Signaling Technology, 9661), Anti-mouse (Multi Sciences, GAM0072) and anti-rabbit (Multi Sciences, GAR0072). ECL Chemiluminescent Kit (Thermo Fisher, 03781) was used to visualize the antibody-antigen interaction and chemical luminescence of membranes was detected by Amersham Imager 600 (GE).

### Animal and cancer cachexia mouse model

All animals care and experimental protocols for this study complied with Chinese regulations and the *Guide for Care and Use of Laboratory Animals* drawn up by the National Institutes of Health (United States) and were approved by the Institutional Animal Care and Use Committee of the East China Normal University (m20210104). The animal experiments were designed and accomplished, as previously described [6]. Male BALB/c mice (6–8 weeks) for inoculation of C26 tumor cells or male C57BL/6 mice (6-8 weeks) for inoculation of MC38 tumor cells were purchased from the Shanghai SLAC Laboratory Animal Co., Ltd and Shanghai Jihui Laboratory Animal Care Co., Ltd respectively. All mice were maintained on a 12:12-h light-dark cycle in a temperature-controlled (21–23 °C) and specific pathogen-free (SPF) conditional room and were provided standard rodent chow and water *ad libitum*. Animals were acclimatized for a week before beginning the study. BALB/c mice were randomly divided into two groups: the healthy group (without tumor, *n* = 8), and the C26 tumor-bearing mice (C26 TB, *n* = 8). No blinding was done. There are no inclusion/exclusion criteria. At the beginning day (D0), C26-TB mice were implanted subcutaneously in the right flank with 0.1 mL (1 × 10^6^) of C26 cells, and the mice in healthy group were implanted subcutaneously in the right flank with isometric sterile PBS. C57BL/6 mice were randomly divided into two groups: the healthy group (without tumor, *n* = 8), and the MC38 tumor-bearing mice (MC38 TB, *n* = 8). No blinding was done. There are no inclusion/exclusion criteria. At the beginning day (D0), MC38-TB mice were implanted subcutaneously in the right flank with 0.1 mL (2 × 10^6^) of MC38 cells, and the mice in healthy group were implanted subcutaneously in the right flank with isometric sterile PBS. Body weight, tumor volume and body temperature were measured daily from inoculation to completion of the study. The shortest diameter (X) and the longest diameter (Y) of tumor were measured with calipers. Tumor volume was calculated following the formula V = X*X*Y*0.5. When the mice body weight lost 10% or when their tumor volume reached 2000 mm^3^, the experiment was terminated. The tumor tissues and GA muscle tissues were rapidly dissected, weighed and then used for further analysis. No blinding was done to the researcher who conducted the measurements and outcome assessments.

### Immunofluorescent staining

The immunofluorescent staining and quantitative assessment of myotubes were conducted, as previously described [6]. At the end of experiments, the C2C12 myotubes were washed three times with phosphate-buffered saline (PBS) and then fixed in 4% PFA for 30 min at room temperature, permeabilized with 0.5% Triton X-100 in PBS for 10 min, and then blocked with 5% BSA in PBS for 1 h at room temperature. Myotubes were incubated with anti-MHC (MF-20, 1:100, DSHB) diluted in 5% BSA overnight at 4 °C. Myotubes were incubated with secondary antibody Cy3-AffiniPure rabbit anti-mouse IgG (H + L) (1:500, Jackson) at room temperature. Images were captured by fluorescence microscope (Leica) and the diameter of myotubes was quantified on images using Image J software. Briefly, 10 fields were randomly selected to determine the average diameters of at least 100 myotubes for each condition. Results of three independent experiments were used for statistical analysis.

### Statistical analysis

Data are expressed as mean ± SEM. Two-tailed Student’s *t* test was used for comparisons between two groups. All analyses were performed using GraphPad Prism 6.0. Values of *p* less than 0.05 were considered to be statistically significant and were presented as *p < 0.05, **p < 0.01, ***p < 0.001. No statistical method was used to pre-determine sample size. Normal distribution of data and data variation was not assessed.

## Supplementary information

Supplemental Material

CDDISCOVERY-21-2870-reproducibility checklist

## Data Availability

The data presented in this study are available on request from the corresponding author. The data are not publicly available due to privacy.
