# Supplementary material for: GDF-15 in tumor-derived exosomes promotes muscle atrophy via Bcl-2/caspase-3 pathway
Source: Cell Death Discov. 2022 Apr 4;8:162. doi: 10.1038/s41420-022-00972-z (PMC8980041; doi:10.1038/s41420-022-00972-z)

Original Western Blots

Figure 1C

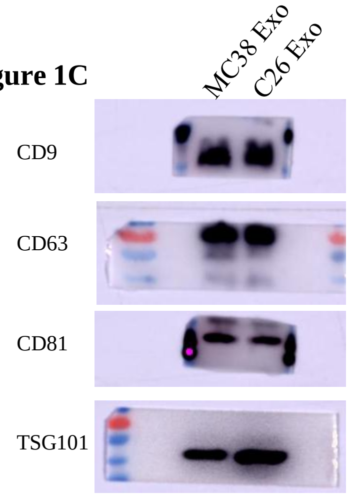

Figure 1F

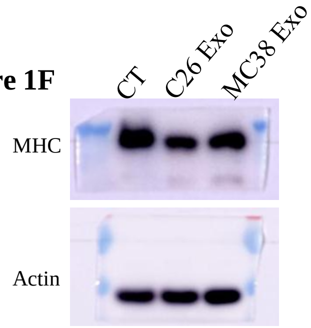

Figure 2A

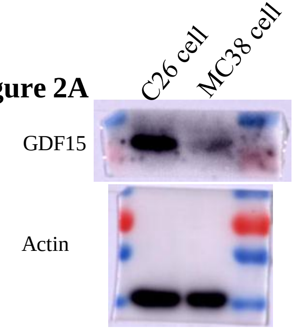

Figure 2B

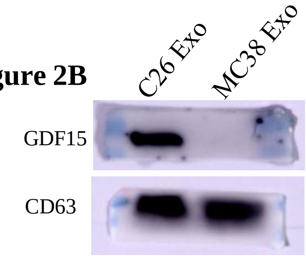

Figure 2C

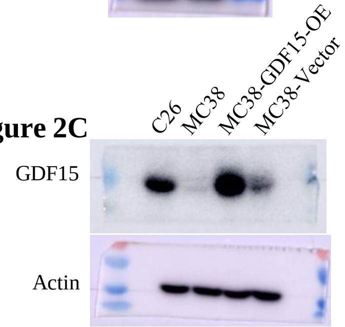

Figure 2D

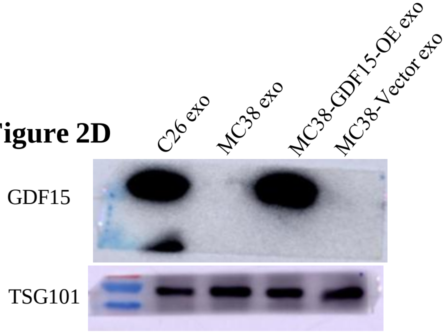

Figure 2E

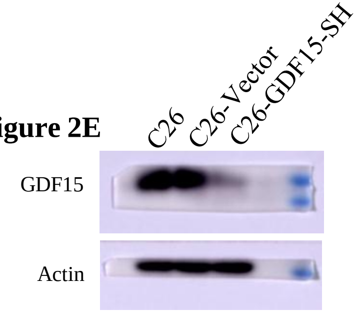

Figure 2F

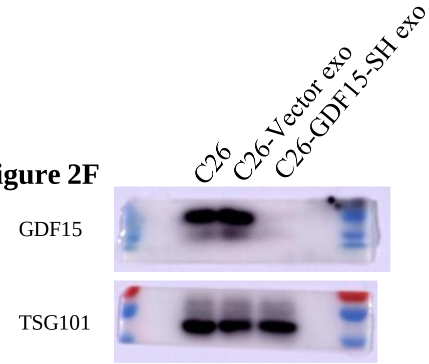

Figure 2G

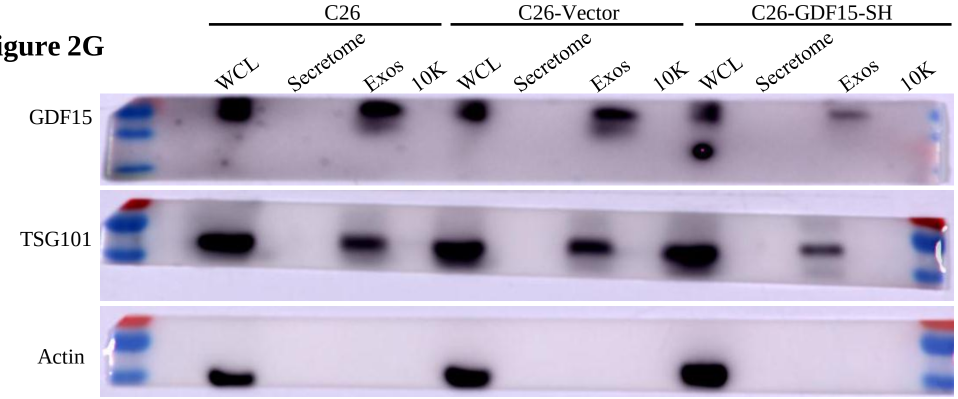

Figure 2I

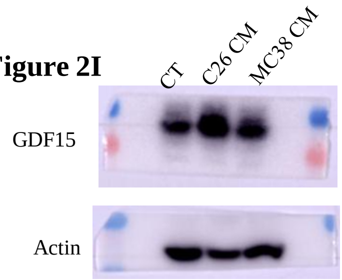

Figure 2H

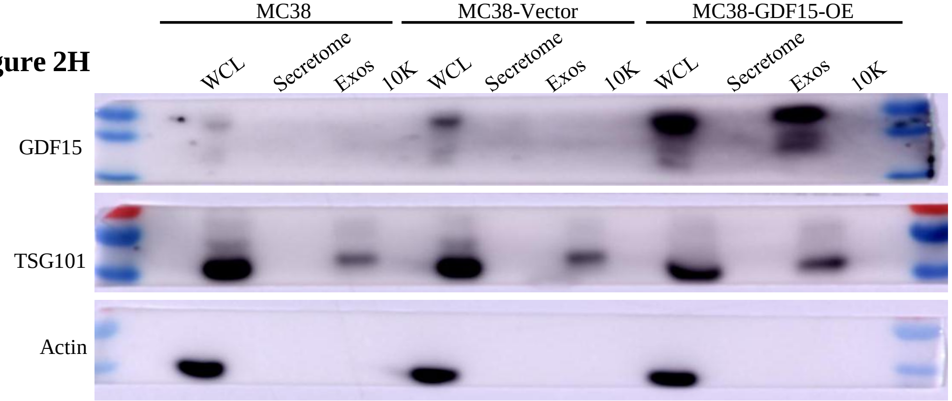

Figure 2J

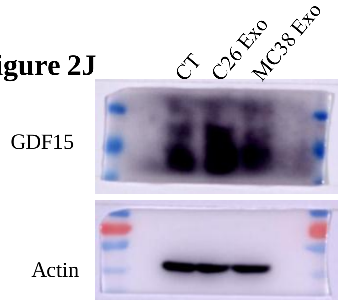

Figure 3C

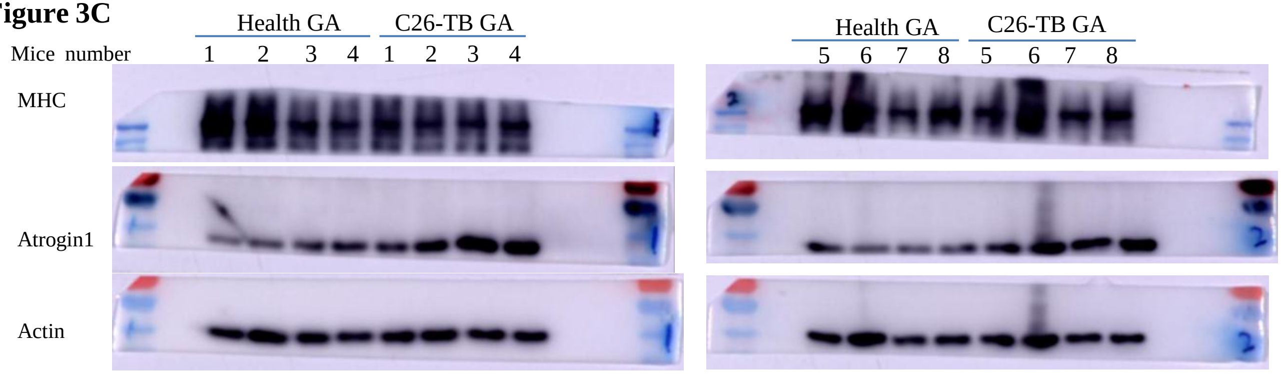

Figure 3E

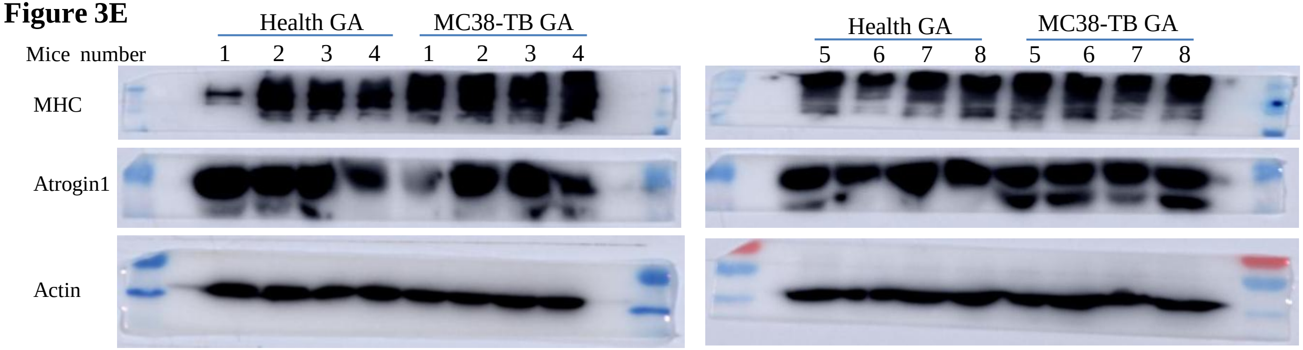

Figure 3G

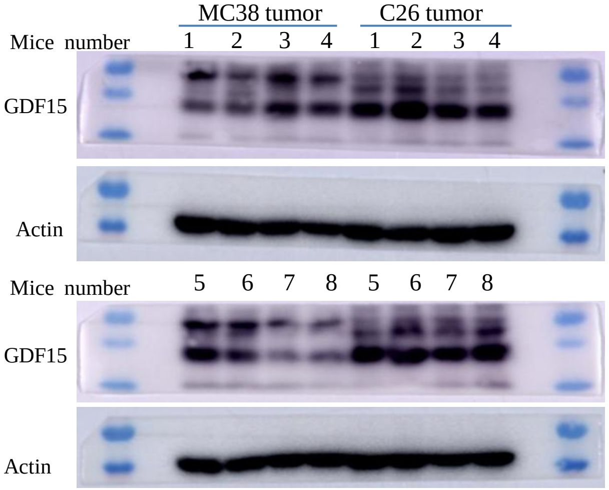

Figure 3I

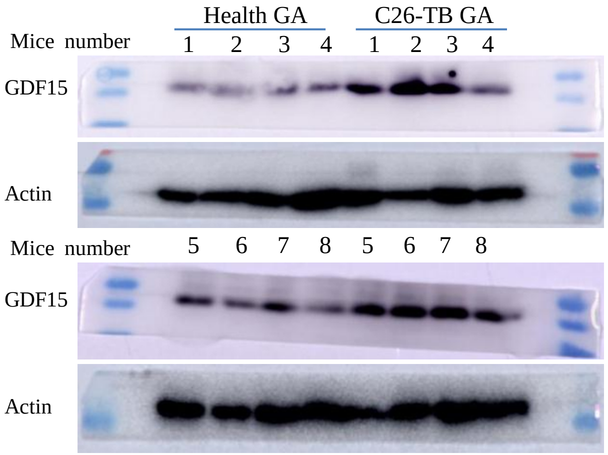

Figure 3H

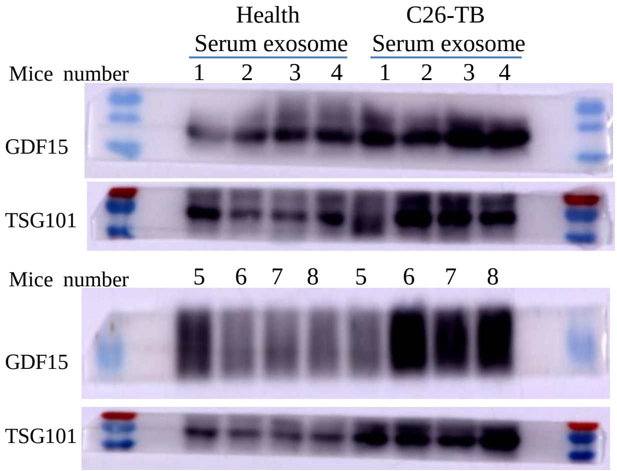

Figure 3J

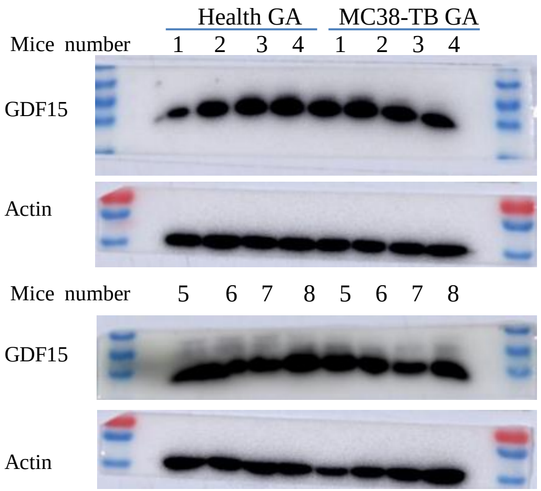

Figure 4C

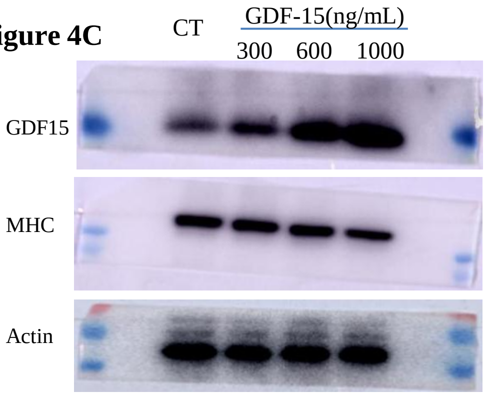

Figure 4F

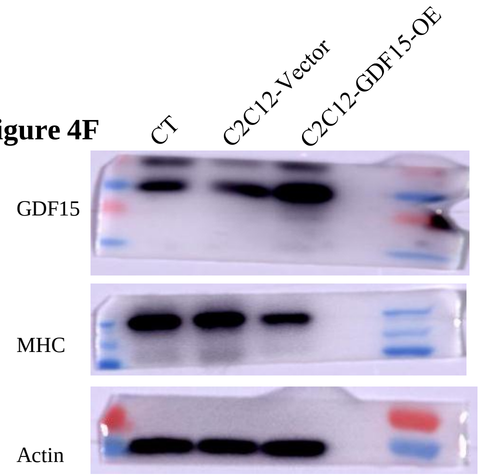

Figure 5A

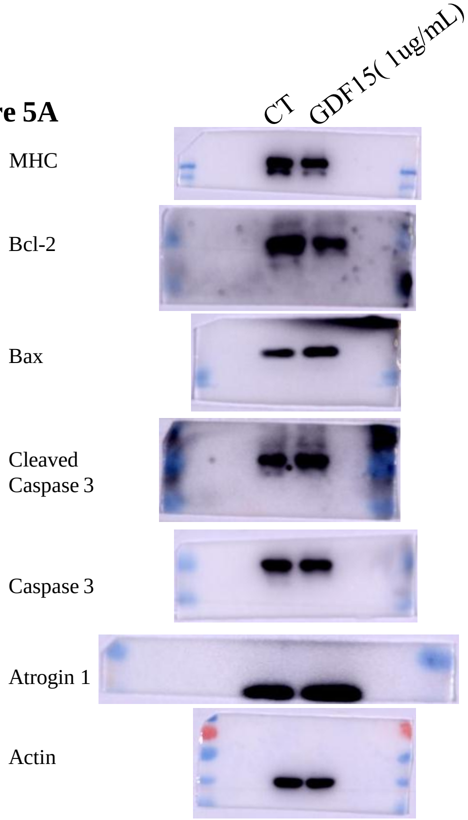

Figure 5B

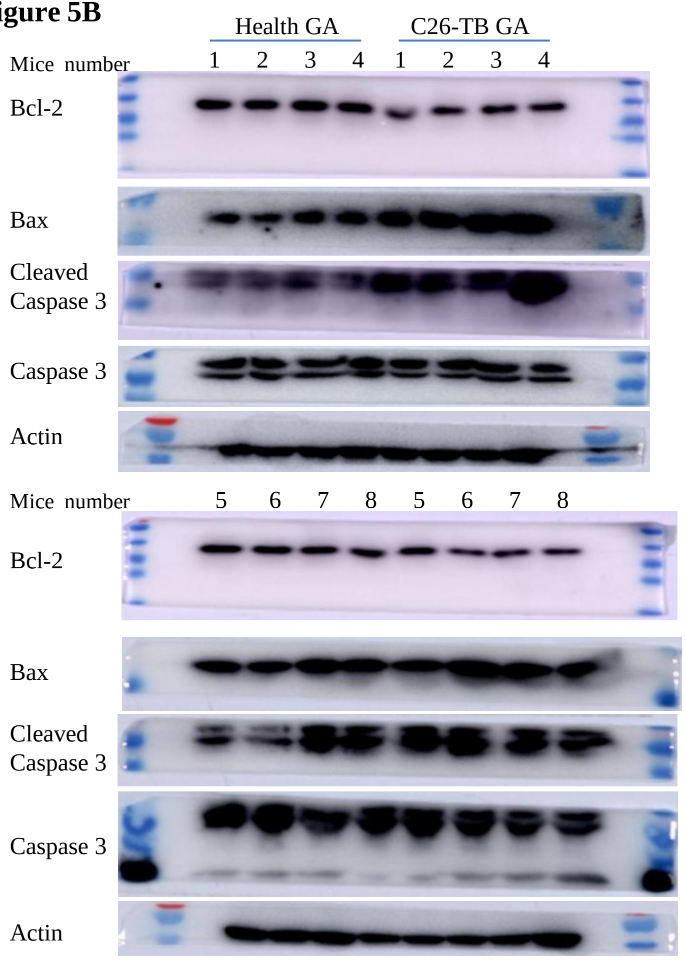

Figure 5C

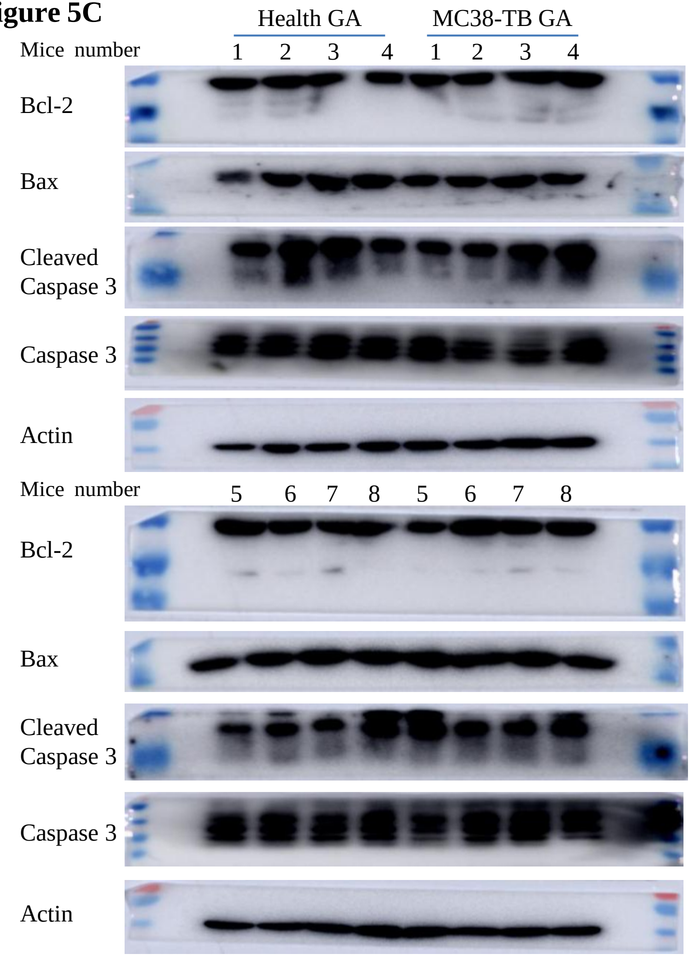

Figure 6C

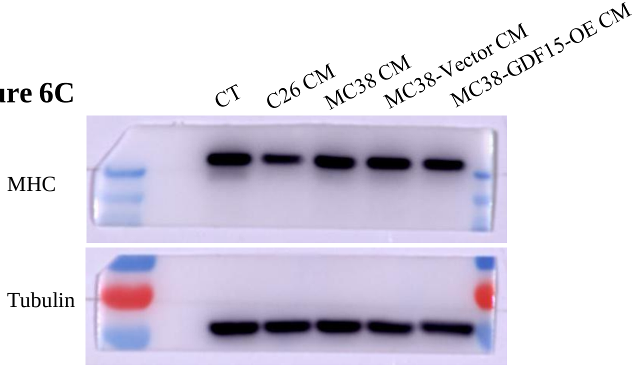

Figure 6I

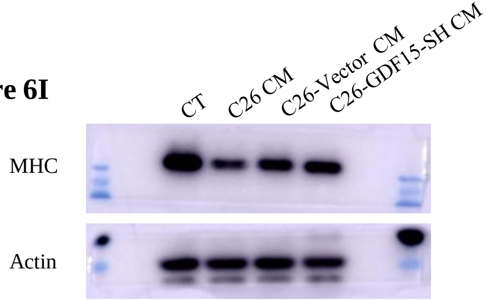

Figure 6F

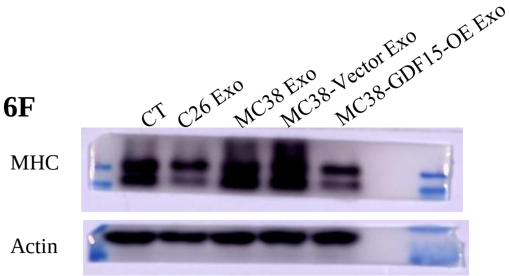

Figure 6L

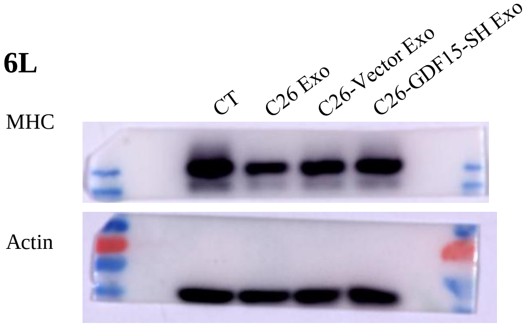

Figure 7A

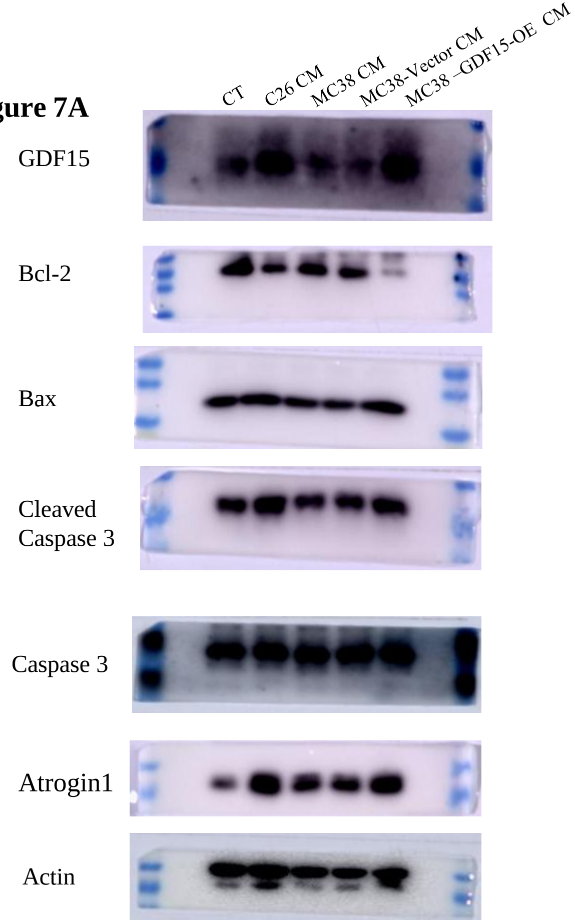

Figure 7B

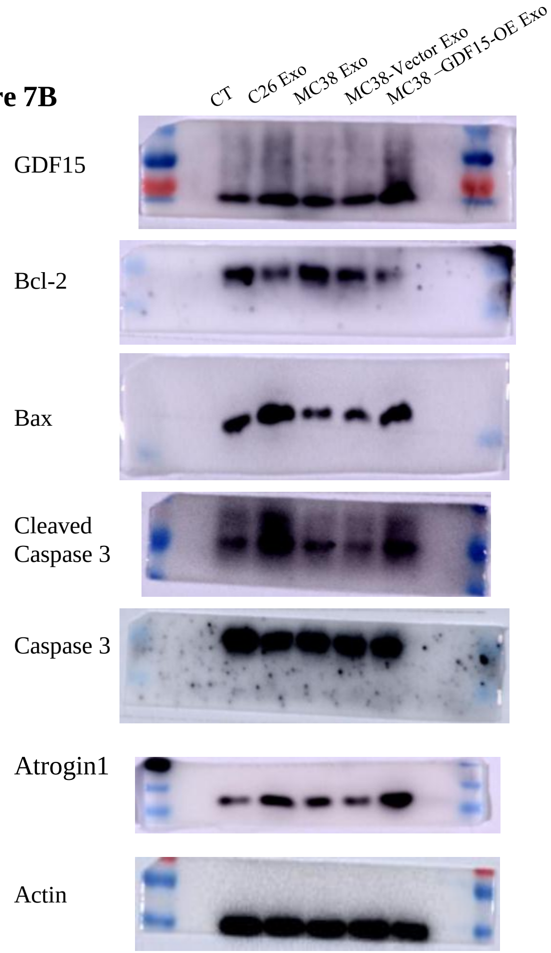

Figure 7C

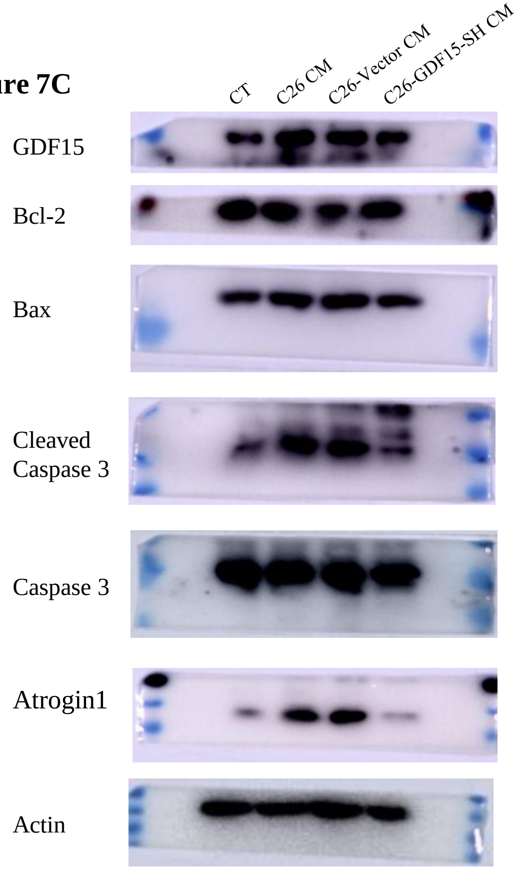

Figure 7D

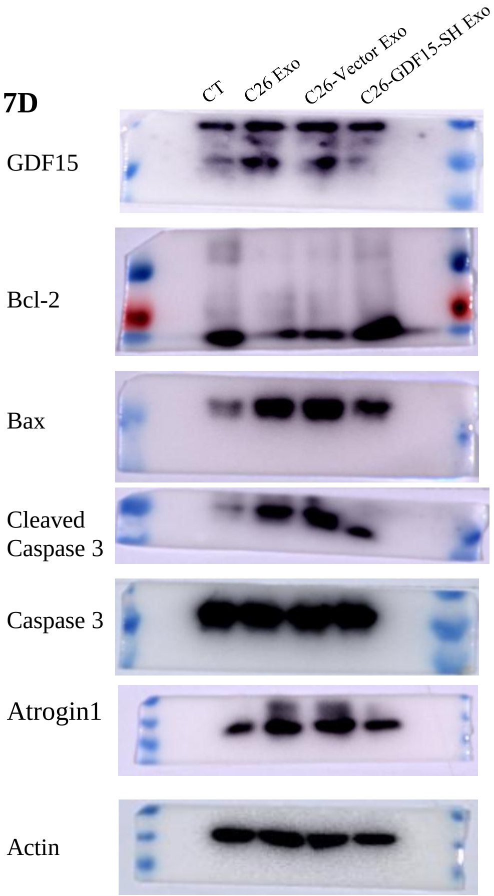

Supplement: Supplementary file 1 — Supplemental Material [file 41420_2022_972_MOESM1_ESM.pdf]
